# The Synaptic Scaling Literature: A Systematic Review of Methodologies and Quality of Reporting

**DOI:** 10.3389/fncel.2020.00164

**Published:** 2020-06-16

**Authors:** Thiago C. Moulin, Danielle Rayêe, Michael J. Williams, Helgi B. Schiöth

**Affiliations:** ^1^Institute of Medical Biochemistry Leopoldo de Meis, Federal University of Rio de Janeiro, Rio de Janeiro, Brazil; ^2^Functional Pharmacology Unit, Department of Neuroscience, Uppsala University, Uppsala, Sweden; ^3^Institute of Biomedical Sciences, Federal University of Rio de Janeiro, Rio de Janeiro, Brazil; ^4^Department of Ophthalmology and Visual Sciences, Albert Einstein College of Medicine, New York, NY, United States; ^5^Institute for Translational Medicine and Biotechnology, Sechenov First Moscow State Medical University, Moscow, Russia

**Keywords:** synaptic scaling, homeostatic plasticity, systematic review, molecular methods, electrophysiology, risk of bias assessment, quality of reporting

## Abstract

The maintenance of the excitability of neurons and circuits is a fundamental process for healthy brain functions. One of the main homeostatic mechanisms responsible for such regulation is synaptic scaling. While this type of plasticity is well-characterized through a robust body of literature, there are no systematic evaluations of the methodological and reporting features from these studies. Our review yielded 168 articles directly investigating synaptic scaling mechanisms, which display relatively high impact, with a median impact factor of 7.76 for the publishing journals. Our methodological analysis identified that 86% of the articles made use of inhibitory interventions to induce synaptic scaling, while only 41% of those studies contain excitatory manipulations. To verify the effects of synaptic scaling, the most assessed outcome was miniature excitatory postsynaptic current (mEPSC) recordings, performed in 71% of the articles. We could also observe that the field is mostly focused on mechanistic studies of the synaptic scaling pathways (70%), rather than the interaction with other types of plasticity, such as Hebbian processes (4%). We found that more than half of the articles failed to describe simple features, such as regulatory compliance statements, ethics committee approval, or statements of conflict of interests. In light of these results, we discuss the strengths and pitfalls existing in synaptic scaling literature.

## Introduction

Animal models are valuable tools for understanding human diseases and physiological mechanisms. However, their application is limited, as just a fraction of the efficacious interventions seems to be translatable to humans (O'Collins et al., 2006). Thus, structured methods of literature synthesis are required to make an objective sense of the large volume of preclinical research and locate the most promising findings. Systematic reviews and meta-analyses are useful tools that can address some of these challenges by providing an objective summary of scientific articles, appraising available evidence, and evaluating the likelihood that a given conclusion is biased (Macleod et al., 2015). For such reasons, the number of systematic studies from preclinical data has been rising in recent years (Vesterinen et al., 2014), mostly focusing on the application of animal models (Sena et al., 2014).

Synaptic scaling is a type of homeostatic plasticity that was first described around 20 years ago (Turrigiano et al., 1998), believed as necessary for proper development and function of neuronal networks (Turrigiano and Nelson, 2004). It is a negative feedback response mechanism to chronic changes in the level of network activity, in which the synaptic strengths of a neuron are modified by regulating synaptic receptors following a universal multiplicative scaling factor. This adjustment happens in a way that the total synaptic input matches the neuron's homeostatic range while preserving the relative differences between synaptic weights (Abbott and Nelson, 2000; Turrigiano, 2012). By a bidirectional interaction with other types of plasticity, it is able to maintain many aspects of neural function and to regulate future synaptic modifications (Fernandes and Carvalho, 2016; Keck et al., 2017a; Moulin et al., 2019).

Moreover, homeostatic plasticity has been shown to influence the pathophysiology of several neuropsychiatric and neurologic disorders, such as intellectual disability (Soden and Chen, 2010), Rett syndrome (Qiu et al., 2012), schizophrenia (Dickman and Davis, 2009), and Alzheimer's disease (Yamamoto et al., 2015). However, despite its relevance, to the best of our knowledge there is no systematic approach to answer questions such as what is impact and reliability of the field, which are the most commonly used techniques, and how the methods are changing over time.

In this study, we performed a systematic review of articles on synaptic scaling to address these issues. Our first goal was to describe important features of the field, such as impact factor distribution and countries where these studies are produced. We investigated which are the popular models for synaptic scaling experiments, followed by an evaluation of the main intervention types to induce homeostatic changes and which outcomes are assessed. We then analyze the reporting of measures to reduce the risk of bias in these studies. We conclude with a discussion on the implications of this research, as well as gaps in the empirical results that limit our understanding of this homeostatic mechanism.

## Methods

### Search Strategy

We performed two separate searches in PubMed to find publications related to synaptic scaling and homeostatic plasticity. Our first search used the most established keywords for describing this process (“homeostatic plasticity” OR “synaptic scaling”), which returned 664 articles. We then performed a second search for articles that might have been missed by those specific keywords, combining the most common descriptions of outcomes and methods (“(mEPSC^*^ OR mIPSC^*^ OR patch clamp^*^) AND (scaling OR homeostat^*^ OR chronic^*^ inhibit^*^ OR chronic^*^ excitat^*^) NOT review”), which returned 618 studies. Duplicated articles (61) were removed. There were no time constraints on the searches, which were both performed on May 31st, 2018.

### Study Selection

The first screening step considered only titles and abstracts, excluding (i) articles not written in English, (ii) articles not presenting original results, such as reviews, and (iii) articles not describing animal experiments using chronic stimulation or inhibition of neurons to study homeostatic synaptic scaling plasticity. This first step was performed by both authors using the Abstrackr online platform (Wallace et al., 2012), and at least one had to include the reference for it to be taken to the next screening stage. If the title and abstract were not clear about the three criteria described above, articles were still included for further screening.

The second screening stage considered the full text of the articles. They were included if they meet the following criteria: (i) described the effects of chronic neuronal stimulation or inhibition on an outcome, (ii) controlled for intensity and time, (iii) used interventions with known effects on synaptic transmission and/or firing of the studied neuronal population, and (iv) investigated changes in neuronal excitability through synaptic homeostatic plasticity, as defined by the objectives and discussion of the article. Despite the subjectivity that is inherent to interpreting phenomena as being due to scaling, our goal was to have a representative sample of the synaptic scaling literature, rather than performing an extensive pursuit of other findings that might correspond to synaptic scaling. After evaluation on these criteria, we used the included articles to extract the type of experiments performed, the study and journal citation metrics, and the reporting of measures to control the risk of bias. At this stage, data for each article were extracted by one of the authors.

### Data Extraction

We built Microsoft Excel spreadsheets as a database to include all articles selected in the first screening stage. For those that met inclusion criteria, data obtained from the second screening stage were also stored in this database. The following items were extracted and recorded for the systematic review:

Publication features: PMID; first author's name; journal name; year of publication; country of origin (defined by the corresponding author affiliation); and impact factor of the journal (obtained from the Scimago Journal Rank for the publication year).

Risk of bias assessment: Blinded assessment of outcome; unbiased methods for data selection (the description of any method aiming to diminish the possible bias occurring in data selection, e.g., randomly selecting 10 out of 100 mEPSC recordings to analysis); the presence of sample size or power calculation within the article; statement regarding compliance with regulatory requirements for animal research; statement of local ethics committee approval; statement regarding conflict of interest on the part of the authors. These items were considered present if they were described at any point in the article.

Experimental features: “Direct / Indirect” intervention—whether the article performed a manipulation directly on the neuronal population later assessed for scaling (e.g., assessment of neurons chronically treated with TTX), or on a circuit projecting to the neuronal population tested for scaling, (e.g., monocular deprivation with visual cortex assessment, or entorhinal denervation with hippocampal DG recordings). “Intervention method”—description of the substance(s) or method(s) of intervention used to induce scaling (e.g., TTX, ChR2, visual deprivation). “Species”—the animal species used in the experiments. “*In vitro*/*in vivo*” and “Model application”–brief descriptions of the model used in the experiments to induce synaptic scaling (e.g., *in vitro* hippocampal primary culture). “Inhibitory / Excitatory” interventions—the presence of inhibitory or excitatory manipulations to induce synaptic scaling. “mEPSC”—the presence of miniature excitatory post-synaptic current recordings. “mIPSC”—the presence of miniature inhibitory post-synaptic current recordings. “Dendritic spines”—the presence of an assessment of dendritic spine density or area. “Synaptic membrane channels”—the presence of an assessment of the transcription or expression of synaptic membrane channels/receptors or their subunits. “Other synaptic proteins”—the presence of an assessment of other synaptic proteins (e.g., PSD95, GAD65, VIAAT). “Effect on Hebbian plasticity”—the presence of an assessment of Hebbian plasticity (e.g., induction of LTP or LTD) after synaptic scaling protocols. “Interference with scaling mechanism”—the presence of experiments studying the effects of interfering with specific mechanisms on scaling (e.g., using pharmacological or genetic interventions to identify the pathways involved in the synaptic scaling). “Firing rate homeostasis”—the presence of neuronal spiking assessment to evaluate the homeostatic effects of the manipulation in the neuronal function. “Multiplicative scaling”—whether the article discusses multiplicative scaling changes in the mPSC amplitudes, and if it is demonstrated by linear fit/ regression of the ranked mPSC amplitude distributions, or by performing the Kolmogorov-Smirnov test after multiplying the cumulative amplitude distribution by a scaling factor.

## Results

### Article Selection and Inclusion

**Figure 1:**
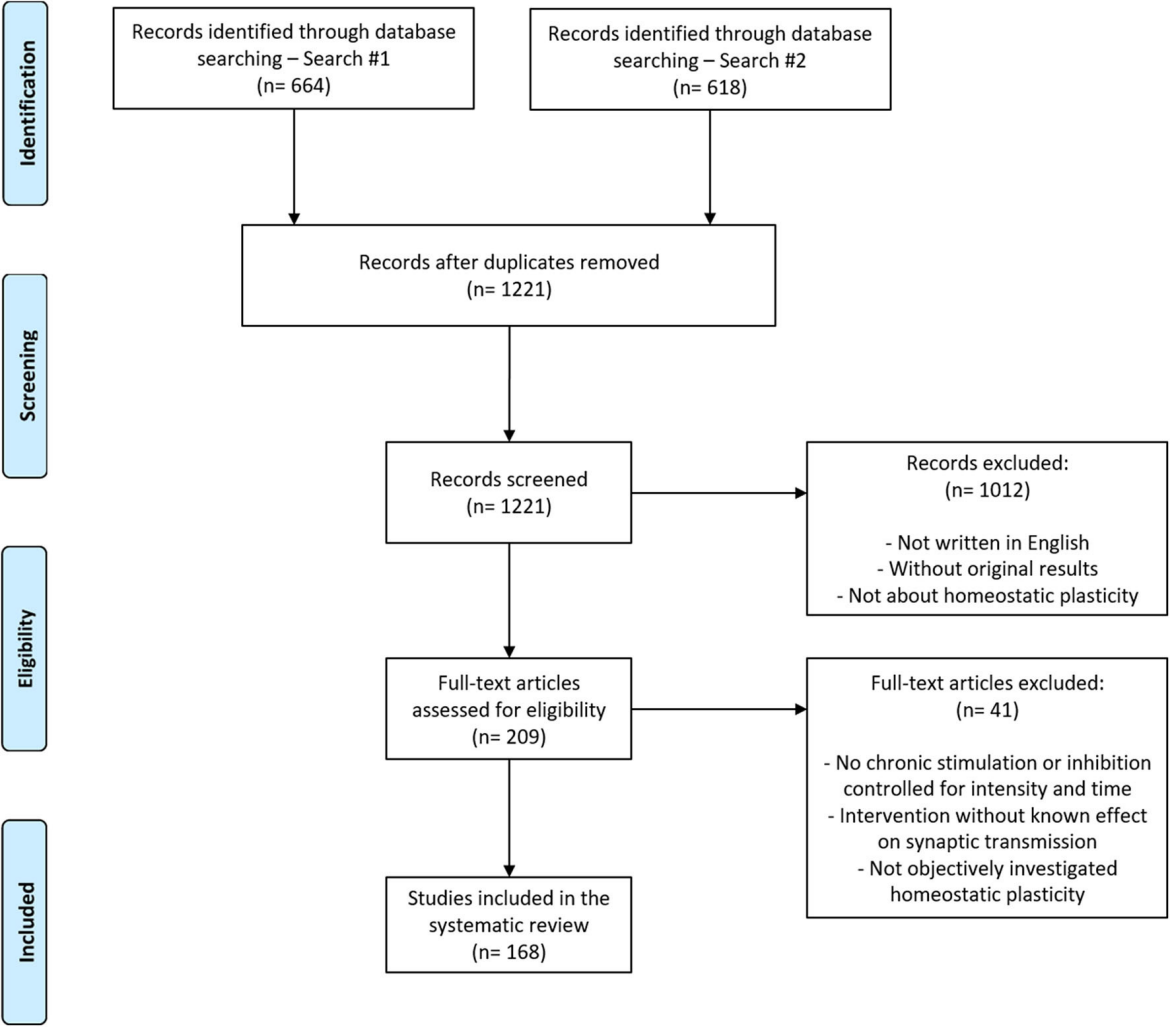
Flowchart of article search and selection. Of the 1,221 articles retrieved from the combination of two search strategies, 168 were included in our analysis after the two-stage screening process (see section Methods for details).

Articles were screened by combining two search strategies to broaden the detection of relevant studies (see Methods). After the exclusion of duplicates, 1,221 articles were obtained (Figure 1). In the first screening step, two investigators examined all articles based on titles and abstracts, and the agreement for exclusions measured on a double-screened sample of 200 articles was 95%. It led to 209 articles selected for full-text screening. Ultimately, 168 articles met all criteria and were considered for further analysis.

### Literature Characteristics

**Figure 2:**
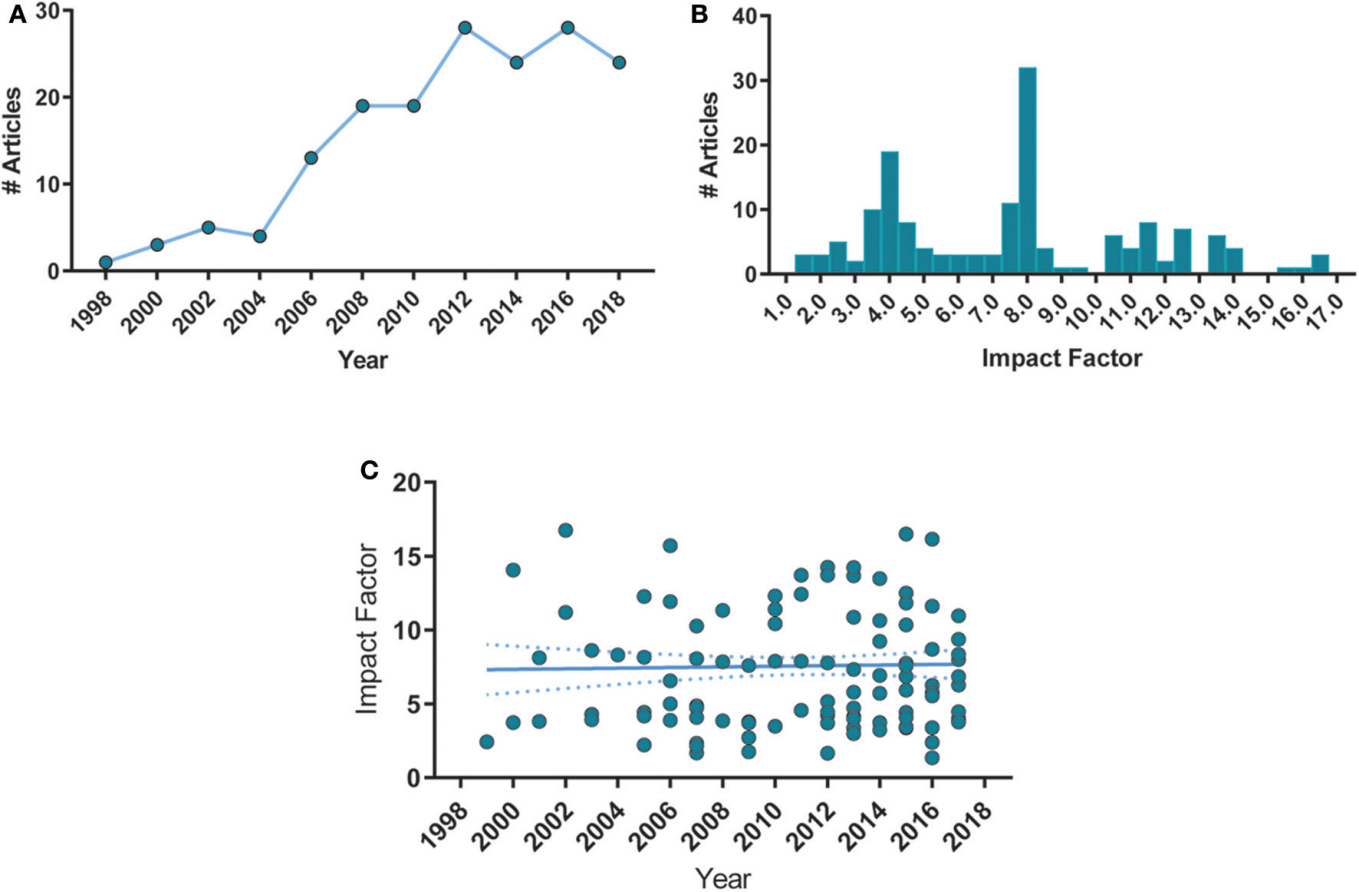
Histogram distributions of articles per publication year and impact factor. **(A)** Number of article publications over time. Each point corresponds to a 2-year bin. Spearman's correlation, ρ = 0.93, *p* < 0.0001. **(B)** Number of articles distributed by their respective journals' impact factor, with a bin size of 0.5. Median = 7.76, min = 1.36, max = 16.74, *n* = 157. **(C)** Mean impact factor remained stable over time. Spearman's ρ = −0.001, *p* = 0.904. Solid lines represent the linear fit of the data. Dashed lines are the 95% C.I. of the linear fit.

First, we analyzed the year of publication of all articles and the distribution of impact factors of their respective journals (Figure 2). Impact factors (number of citations divided by the number of citable documents for the previous 2 years) were obtained through the Scimago Journal Rank database corresponding to the year of publication and were unavailable for 11 of the included articles. There was a significant increase in publications over the years (Figure 2A), with a median impact factor of 7.76 (Figure 2B). Additionally, we noticed that the mean impact factor over the years remained stable (Figure 2C). These results suggest that the interest of high-impact journals on the subject has remained elevated over the years. Regarding demographics, more than 80% of studies from our sample were originated from the United States, Germany, and the United Kingdom (Supplementary Table 1).

### Features of the Experimental Models

Next, we investigated which animal models were mostly employed for synaptic scaling studies, either by the use of the whole organism during *in vivo* trials or as the tissue source for *in vitro* experiments. Rodents were the most prevalent species, as rats were used in 52% of the studies, followed by nearly 40% of the reports employing mice. Interestingly, there is a significant decrease in rat-base testing over time, while the usage of mice significantly grew over the years.

Moreover, *in vitro* models seem to be the approach of choice for the field (83%), largely due to experiments using dissociated-cell cultures, present in almost 60% of our sample, followed by organotypic cultures (18%). For the articles with *in vivo* investigations (18%), most were performed by sensorial manipulations (13%), while direct circuit interventions (e.g., pharmacological or optogenetic stimulation in a given brain area) were present in only 5% of our sample.

### Methodological Aspects of Synaptic Scaling Assessment

We analyzed the main experimental features from the sample articles regarding protocols to induce and evaluate synaptic scaling (Table 2). We first categorized different kinds of scaling-inducing interventions as excitatory (e.g., bicuculline, picrotoxin) or inhibitory (e.g., TTX, visual deprivation). We also classified the interventions as direct (i.e., applied directly to the neuronal population assessed for homeostatic changes) or indirect (i.e., applied to a pre-synaptic circuitry from the studied neurons). The vast majority of the articles (86%) employ inhibitory interventions to induce synaptic scaling, while less than half of the studies (41%) contain excitatory ones. The most popular inhibitory manipulation was TTX, used in 55% of the articles, while bicuculline was the most used intervention for neuronal excitation (26%). A list of the main manipulations used in the studies can be found on Supplementary Table 2. We observed that these interventions are mostly administered directly to the same neurons from which the scaling outcomes are measured (89%), rather than indirectly via other circuits or sensorial systems (12.5%).

**Table 1:** Experimental models.

**Species**	**# Articles (%) [95% C.I]**	**Trend over time (ρ)**	***p*-value**
Mouse	66 (39.3) [32.2, 46.8]	0.225	0.0034^#^
Rat	87 (51.8) [44.3, 59.2]	−0.283	0.0002^#^
Drosophila	7 (4.2) [2.0, 8.3]	0.172	0.026
Chicken	4 (2.4) [0.9, 5.9]	0.048	0.533
Others	5 (3.0) [1.3, 6.8]	−0.019	0.808
Not described	2 (1.2) [0.3, 4.2]	−0.086	0.263
**Model application**			
*In vitro*	140 (83.3) [76.9, 88.2]	0.011	0.888
Dissociated-cells culture	99 (58.9) [51.3, 66.1]	0.009	0.909
Organotypic culture	31 (18.4) [13.3, 25.0]	−0.116	0.135
Acute brain slice	4 (2.4) [0.9, 5.9]	−0.035	0.651
Others	8 (4.7) [2.4, 9.1]	0.122	0.114
*In vivo*	31 (18.4) [13.3, 25.0]	0.021	0.791
Sensorial manipulations	22 (13.1) [8.8, 19.3]	0.053	0.496
Brain circuitry intervention	9 (5.4) [2.6, 10.2]	−0.044	0.573

*The columns show the number of articles reporting each item, with percentages relative to the total number of articles included (n = 168 for all items), and their 95% confidence intervals. Spearman's correlation was used to estimate the ρ coefficient and p-values for model use over time*.

# *Significantly correlated with time (α = 0.0085 for species correlations and α = 0.0064 for model application correlations, Bonferroni correction for multiple comparisons)*.

**Table 2:** Intervention and assessment features.

**Intervention to induce scaling**	**# Articles (%) [95% C.I]**	**Trend over time (ρ)**	***p*-value**
Inhibition	145 (86.3) [81.1, 91.5]	−0.061	0.435
Excitation	69 (41.1) [33.7, 48.5]	0.046	0.558
Direct	149 (88.7) [83.9, 93.5]	−0.070	0.370
Indirect	21 (12.5) [7.5, 17.5]	0.073	0.344
**Outcome evaluated**			
mEPSCs	120 (71.4) [64.6, 78.2]	0.115	0.137
mIPSCs	25 (14.9) [9.5, 20.2]	−0.089	0.253
Dendritic spines	13 (7.7) [3.7, 11.8]	0.024	0.756
Synaptic channels	67 (39.9) [32.5, 47.3]	0.036	0.646
Other synaptic proteins	27 (16.1) [10.5, 21.6]	−0.128	0.098
**Additional features**			
Interference with scaling mechanism	118 (70.2) [63.3, 77.1]	0.314	<0.0001^#^
Effect on Hebbian plasticity	7 (4.2) [1.2, 7.2]	0.066	0.394
Firing rate homeostasis	40 (23.8) [1.8, 30.8]	−0.097	0.212
Multiplicative scaling	49 (29.2) [22.5, 36.8]	−0.018	0.815

*The number of articles that contains a reported item, with the percentages relative to the total quantity of articles included (n = 168), and the 95% confidence interval is shown. Spearman correlation test was used to estimate the ρ coefficient and p-values for the application of the methods over time*.

# *Significantly correlated with time (α = 0.0046 after Bonferroni correction for multiple comparisons)*.

Next, we assessed the widespread outcomes tested after inducing synaptic scaling, such as miniature excitatory and inhibitory postsynaptic currents (mEPSCs/ mIPSCs), present in 71% and 15% of the reports, respectively; analyses of dendritic spines (density, area, or volume) (8%); and relative changes in synaptic channels (40%) or other synaptic proteins (16%).

To investigate the number of reports that consider the specific components of synaptic scaling, we registered whether the articles had protocols for interfering with mechanisms or pathways of scaling processes (e.g., inhibition of a given transcription factor to study its effects) (70%); if they studied the influence of homeostatic plasticity on Hebbian-like mechanisms (e.g., by inducing LTP or LTD after scaling protocols) (4%); and the assessment of hallmark characteristics, such as whether firing rate homeostasis is observed (24%) or if the changes in mPSC follow multiplicative changes (29%).

The description of methods for analyzing multiplicative scaling was also evaluated (Supplementary Figure 1). Within the articles with this feature, performing a Kolmogorov-Smirnov test after multiplying the amplitude distribution by a scaling factor was present in 29% of the reports, while linear regression/ correlation analysis for the ranked amplitudes was described in 26% on them. The combined use of these analyses was observed in 33% of the articles.

### Associations Between Experimental Procedures

**Figure 3:**
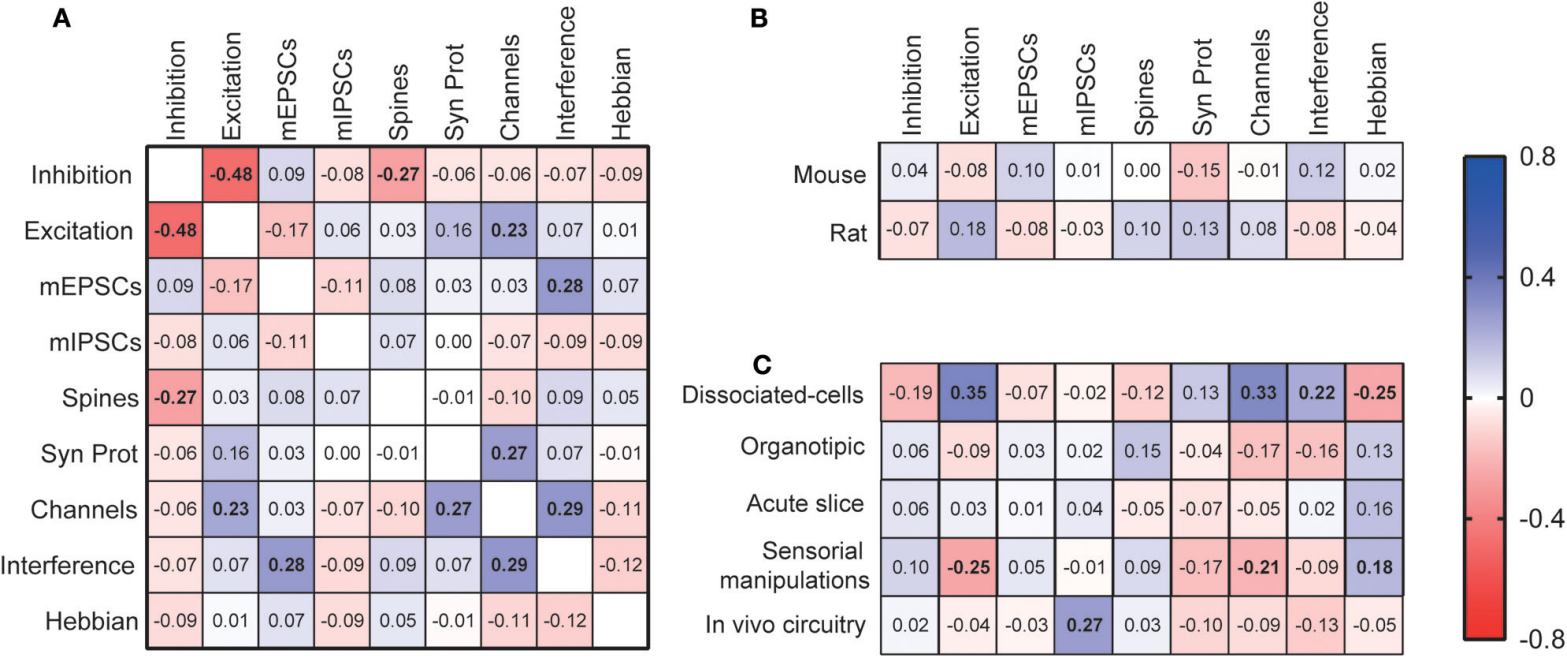
Correlations among experimental features. **(A)** Correlation matrix for the use of different synaptic scaling assessment methods. These features were also correlated with the most used species **(B)** and experimental models **(C)**. “Inhibition”—inhibitory scaling interventions; “Excitation”— excitatory scaling interventions; “Spines”—dendritic spine assessment; “Channels”—quantification of synaptic channels; “Syn Prot”—quantification of other synaptic proteins; “Interference”—manipulations interfering with mechanisms of synaptic scaling; “Hebbian”—investigation of the effects of scaling on Hebbian plasticity. Displayed numbers are the ρ coefficients from Spearman's correlations, which are represented in bold if significantly correlated after Bonferroni correction for multiple comparisons.

We then calculated the correlations between the different methodological aspects of synaptic scaling experiments (Figure 3A). We observed that the reporting of inhibitory interventions to induce scaling has a negative correlation with the reporting of excitatory manipulations (ρ = −0.48, *p* < 0.0001), indicating that most studies are usually limited to one of the approaches. Assessment of dendritic spines tends to be less present when inhibitory interventions are used (ρ = −0.27, *p* = 0.0003), while analyses of synaptic channels or receptors are more common in studies with excitatory interventions (ρ = 0.23, *p* = 0.002). Studies measuring synaptic channels are also more likely to analyze other synaptic proteins (ρ = 0.27, *p* = 0.0004). Also, articles using manipulations interfering with synaptic scaling mechanisms are more likely to report mEPSCs measurements (ρ = 0.28, *p* = 0.0002), and quantifications of synaptic membrane channels (ρ = 0.29, *p* = 0.0001).

We then investigated the relationship between the choice of experimental models and methodology. No significant correlation was found between the use of either mice or rats, the most popular species, and assessment features. However, when analyzing specific experimental models, many methodological preferences were identified. First, dissociated-cell cultures were the most adopted model in studies reporting excitatory manipulations (ρ = 0.35, *p* < 0.0001), when membrane channels were assessed (ρ = 0.33, *p* < 0.0001), and when there were interferences with synaptic scaling mechanisms (ρ = 0.22, *p* = 0.004). However, this model was avoided if the articles were investigating the relationship between Hebbian and synaptic scaling types of plasticity (ρ = −0.25, *p* = 0.001). Studies employing models of sensorial manipulations follow an opposite pattern, as they are less prevalent when articles report excitatory manipulations (ρ = −0.25, *p* = 0.001), or membrane channel measurements (ρ = −0.21, *p* = 0.00), but are preferred when Hebbian plasticity is considered (ρ = 0.18, *p* = 0.006). Finally, when studies induce synaptic plasticity by *in vivo* circuitry manipulations, we observe an increase in the report of mIPSCs evaluations (ρ = 0.27, *p* = 0.0004).

### Risk of Bias Assessment

The description of measures to reduce risk of bias within each study was evaluated by reporting of the following items: blinded assessment of outcomes, unbiased data selection, sample size and/or power calculation, statement of compliance with regulatory requirements, statement of approval by an ethics committee, and statement on conflict of interest (see Methods for definitions of each item). We analyzed the frequency of reporting for each of these items, as well as its correlation with the publication year (Table 3). Our results are comparable with previous studies that described a low incidence of reporting risk of bias measures for animal disease models (Sena et al., 2014; Macleod et al., 2015), and for basic-research paradigms such as fear conditioning (Carneiro et al., 2018). In our sample, the reporting of common features, such as regulatory compliance statement, ethics committee approval, and conflict of interest, was observed in less than half of articles. However, these features showed a significant increase over time, suggesting that the increase of reporting demands, perhaps due to journal policies (McNutt, 2014), is having an impact on this field.

**Table 3:** Risk of bias measures.

**Attribute**	**# Articles (%) [95% C.I]**	**Trend over time (ρ)**	***p*-value**
Blinded outcome assessment	35 (20.8) [14.7, 27.0]	−0.118	0.129
Unbiased data selection	28 (16.7) [11.8, 23.0]	−0.020	0.792
Power or sample size calculation	4 (2.4) [0.9, 5.9]	0.166	0.032
Regulatory compliance statement	77 (45.8) [38.4, 53.3]	0.209	0.007^#^
Conflict of interest*	72 (42.9) [35.6, 50.4]	0.529	<0.0001^#^
Ethics committee approval**	74 (46.0) [38.4, 53.6]	0.387	<0.0001^#^

The first column shows the number of articles reporting each item, with percentages relative to the total number of articles included (n = 168, except for ethics committee approval), and their 95% confidence intervals. Spearman's correlation was used to estimate the ρ coefficient and p-values for reporting trends over time. *Of these, 4 reported an existing conflict of interest. **Seven articles used invertebrate models, which usually do not require the approval of an ethics committee; therefore, the denominator for this item was 161.

# *Significantly correlated with time (α = 0.0083 after Bonferroni correction for multiple comparisons)*.

**Figure 4:**
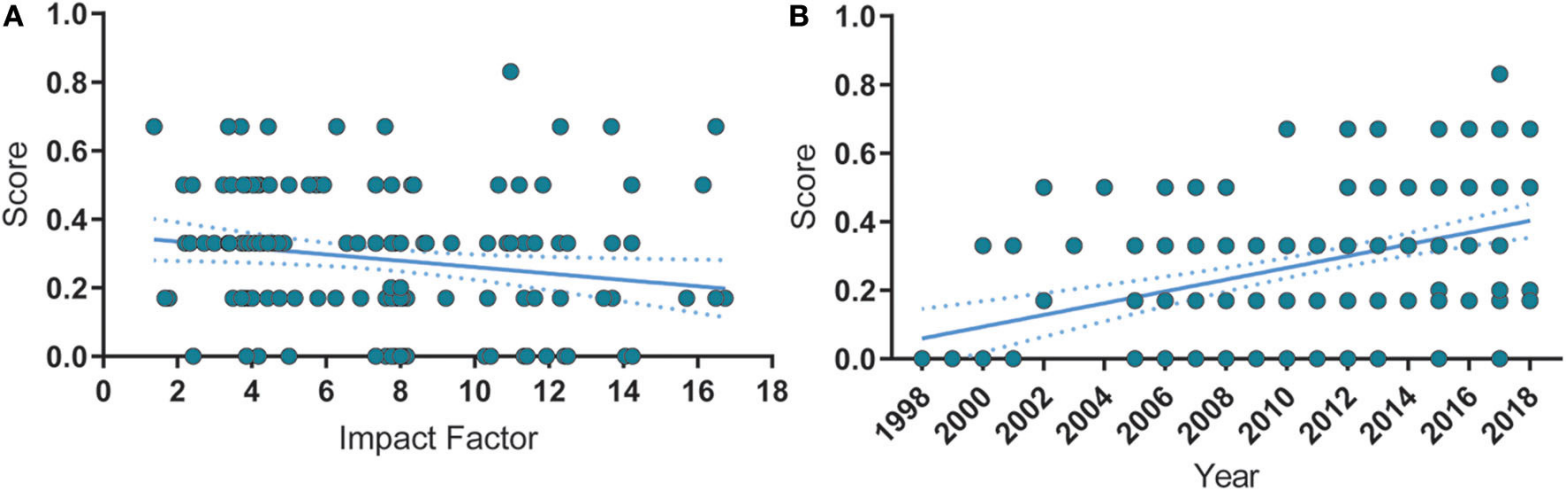
Correlations between reporting score, publication year and impact factor. **(A)** Risk of bias reporting score is negatively correlated with the impact factor. Spearman's ρ = −0.24, *p* = 0.002. **(B)** Over time, the quality score significantly increased. Spearman's ρ = 0.425, *p* < 0.0001. Spearman's ρ = −0.001, *p* = 0.904. Solid lines represent the linear fit of the data. Dashed lines are the 95% C.I. of the linear fit.

Next, we analyze the correlation between overall reporting score (i.e., the fraction of reported risk-of-bias measures) and year of publication or impact factor (Figure 4). Interestingly, the risk of bias reporting score correlated negatively with the impact factor (Figure 4A), although the overall reporting score improved over the years (Figure 4B), indicating that publication in high-impact journals does not safeguard the correct reporting of measures to prevent risk of bias.

## Discussion

The synaptic scaling literature is relatively recent, as the first experimental evidence for this phenomenon was described around 20 years ago (Turrigiano et al., 1998). Nevertheless, we observed a noteworthy number of studies on the subject since then, and a growth in publication volume over the last two decades. The impact factor of the publishing journals has been maintained over time, indicating sustained visibility on the topic. As the systematic reviews of basic-research literature are not usual, such characteristics provide a unique opportunity to compare methods and quality indicators of a relatively new area of basic research to more studied fields, especially applied pre-clinical research.

Our first observation is that the majority of articles in our sample use *in vitro* (83.3%), rather than *in vivo* (18.4%) models, which are mostly based on rodents (51.8% rats, 39.3% mice). This is somewhat expected, as there are many challenges for *in vivo* studies (Lee and Kirkwood, 2019), and *in vitro* experiments would allow for more convenient manipulations for chronic neuronal excitation or inhibition, such as a constant pharmacological administration or direct light stimulation for optogenetics. Accordingly, neuronal cultures were the most popular experimental model (58.9% dissociated cells, 18.4% organotypic). Interestingly, articles reporting experiments employing rats are negatively correlated with time (ρ = 0.283, *p* = 0.0002), while the usage of mice seems to be rising (ρ = 0.225, *p* = 0.0034). This can indicate a shift from the use of rats to mice models, possibly due to the development of genetic manipulations, which are more easily performed in mice (Fahey et al., 2013).

When examining the experimental features of the articles, we observe that most studies have investigated synaptic scaling after chronic inhibition of neuronal activity (86%). Moreover, reporting of inhibitory interventions to induce scaling has a negative correlation with the reporting of excitatory manipulations (ρ = −0.48, *p* < 0.0001). That is a somewhat counterintuitive preference, as the field has long stated the theoretical importance of homeostatic mechanisms for protecting network stability, usually from the effects of excessive activity caused by Hebbian types of plasticity (Abbott and Nelson, 2000; Turrigiano, 2012). Furthermore, the number of empirical studies in our sample about the effects of synaptic scaling on Hebbian-like processes was small (4%), showing that there is some dissonance between theoretical concerns and experimental directions. As many questions on the interaction of these different types of plasticity remain open (Keck et al., 2017b), further research on the topic is required.

The standard practice to demonstrate homeostatic changes is by measurements of parameters of synaptic transmission (i.e., the analysis of presynaptic neurotransmitter release frequency or postsynaptic response amplitude). Accordingly, more than 70% of the studies in our sample assessed synaptic scaling through miniature excitatory postsynaptic currents (mEPSCs), which has been used since the first article describing scaling. On the other hand, miniature inhibitory postsynaptic currents (mIPSCs) were investigated in less than 15% of the articles, which is rather scarce considering that both excitatory and inhibitory currents are thought to be regulated to reach homeostatic activity (Swanwick et al., 2006). Thus, we encourage the investigation of scaling-driven regulation of inhibitory currents in forthcoming studies of the field.

Synaptic scaling can also be explored by examining morphological or molecular markers, such as dendritic spines, synaptic receptors/ channels, and other activity-modulated synaptic proteins. We can observe a correlation in the reporting of measurements of synaptic channels and other synaptic proteins (ρ = 0.27, *p* = 0.0004), suggesting that such morphological parameters are analyzed concomitantly. However, our review shows that these types of experiments are not performed as frequently as the miniature post-synaptic current assessments, indicating that the commonly-accepted demonstrations of scaling-induced changes might be restricted to electrophysiological measurements. We thus believe that the consolidation of alternative parameters, like molecular markers, to confirm the occurrence of synaptic scaling could broaden the experimental range of the field, as it would be more accessible for researchers with different technical expertise.

A large part of the studies uses protocols interfering with homeostatic processes (70%), i.e., using pharmacological or genetic manipulations of specific molecules or cascades to identify those involved in the synaptic scaling. In fact, the only temporal trend found within the experimental features was the growth in the number of such reports over time, suggesting that the field is increasingly focused on the mechanistic description of homeostatic regulation. Our association analysis also showed that these articles are more likely to report mEPSCs measurements (ρ = 0.28, *p* = 0.0002) and quantifications of synaptic channels/ receptors (ρ = 0.29, *p* = 0.0001). Nevertheless, a surprisingly smaller amount of articles investigated fundamental assumptions of synaptic scaling, like its functional role in firing rate homeostasis (24%) or the multiplicative nature of the synaptic changes (29%). Given that post-synaptic currents can be regulated in a non-homeostatic manner (Diering and Huganir, 2018) and that many other types of homeostatic mechanisms do not involve multiplicative adjustments (Keck et al., 2017a; Wang et al., 2019), such assessments are essential for proper identification of scaling-specific processes. Thus, we believe that further attention should be given to confirming the extent of basic scaling features alongside with the employment of homeostatic-inducing interventions.

Moreover, within the articles that mention the multiplicative nature of synaptic scaling, we assessed which ones actually performed statistical tests for its confirmation (Supplementary Figure 1). The most accepted method for determining whether or not multiplicative scaling occurred is based on the analysis of amplitude distributions of the miniature post-synaptic currents (Kim et al., 2012), which can also be applied for correspondent measurements of synaptic puncta, proteins or channels (Keck et al., 2013). First, the recorded amplitudes from the treated cells are rank-ordered and plotted against the rank-ordered control amplitudes. This plot is then fitted with a straight line to obtain the scaling function and, consequently, the scaling factor. Secondly, the individual amplitude values of treated neurons are multiplied by the scaling factor, and a cumulative frequency plot of these amplitudes is constructed. Lastly, the overlap between the treated and control recordings is compared by the Kolmogorov-Smirnov test. Among the 49 articles mentioning multiplicative scaling, 13 (26%) describe employing linear regression of the ranked amplitudes, 14 (29%) report the Kolmogorov-Smirnov comparison analysis of cumulative amplitude distributions, and 16 (33%) describe the whole method with both steps. Interestingly, 6 studies (12%) do not describe any approach for multiplicative scaling assessment, although mentioning this feature in the manuscript. These results indicate that most of articles have a good description of at least of the main steps for multiplicative scaling confirmation.

Regarding the quality of reporting, despite being mainly published in high profile journals, our sample had comparable performance to other areas of animal research in terms of describing procedures to reduce the risk of bias. Less than half of the articles reported basic information such as regulatory compliance statements, ethics committee approval, and conflict of interest. Reporting of blinded outcome assessment was even less frequent and present in only around 20% of the articles. The frequency of reporting for these items was lower than those found in a review of preclinical fields (Macleod et al., 2015) and in a systematic review on fear conditioning (Carneiro et al., 2018). Likewise, sample size or power calculations were performed in a negligible portion of the studies (2.4%).

In addition to these commonly used indicators, we assessed the description of measures to reduce bias in data selection (e.g., randomly selecting mEPSC recordings from a large set; blinding or automatizing the process of selecting images for analysis). To our knowledge, this feature has not been investigated in previous reviews, but as technological advances make it easier to collect large amounts of data on numerous types of experiments, we believe that explicit criteria to select data for analysis are a vital part of a study's methodology. This item was reported in 16.7% of articles in our sample, an encouraging result given the lack of discussion on this topic; however, a value that is still suboptimal for a field highly dependent on extensive data collection.

A commitment to improving *in vivo* research has been stated as a priority by many publishers (McNutt, 2014). Journal demands on conflict of interest disclosures and ethics statements seem to have influenced the synaptic scaling literature, as reporting of these features has significantly increased over time (Table 1). Interestingly, however, the impact factor of the journals is negatively correlated with our risk of bias reporting score in our sample. This diverged from previous reports that have found no statistically significant correlations between these attributes (Macleod et al., 2015; Carneiro et al., 2018). Nonetheless, our sample had a higher median impact factor than the ones analyzed in other reviews, which might indicate that this relation can only be observed in restricted parts of the journal impact factor distribution.

One can argue that high-impact journals have historically imposed strict word count limits, which might have negatively impacted reporting. However, the more recent availability of nearly limitless supplementary data online makes this explanation less likely. There is also evidence that reporting checklists used by high-visibility journals may be less effective than desired: a study that investigated whether journal-requested completion of an ARRIVE checklist improved compliance with the guidelines found little evidence of effectiveness (Hair et al., 2019). Further investigations on the efficiency of journal policies to improve reporting are warranted to broaden this discussion.

## Data Availability Statement

Publicly available datasets were analyzed in this study. This data can be found here: http://doi.org/10.7303/syn21165370.

## Author Contributions

TM conceived and designed the systematic review, prepared the figures, and wrote the manuscript. TM and DR extracted data and performed the statistical analysis. MW and HS contributed with supervision, manuscript editing, and funding acquisition. All authors revised the final version of the manuscript.

## Conflict of Interest

The authors declare that the research was conducted in the absence of any commercial or financial relationships that could be construed as a potential conflict of interest.

## Supplementary Material

Supplementary Figure 1 Types of analysis for multiplicative scaling assessment. From the 168-studies sample, 49 (29%) mention observing multiplicative scaling of mPSC amplitudes. Within articles investigating multiplicative scaling, 14 (28.6%) describe using the Kolmogorov-Smirnov test for comparison of cumulative amplitude distributions, 13 (26.5%) employ linear regression of the ranked amplitudes, and 16 (32.6%) report using both approaches. A number of studies (23) mentioned the use of the Kolmogorov-Smirnov test without assessment of multiplicative scaling, but for simple group-comparison analysis.

Supplementary Table 1 Country affiliation of the corresponding author. Percentages were calculated based on the total number of articles (*n* = 168). If an author had more than one country affiliation, the article counted for both of them; thus, the sum of percentages exceeds 100%.

Supplementary Table 2 Main manipulations to induce synaptic scaling. The table shows the number of articles reporting the use of a given intervention, the percentages relative to the total number of articles included (*n* = 168), and their 95% confidence intervals. Spearman's correlation was used to estimate the ρ coefficient and p values for reporting trends over time. ^#^Significantly correlated with time (α = 0.005 after Bonferroni correction for multiple comparisons).
