# Supplementary figures and images for: The Synaptic Scaling Literature: A Systematic Review of Methodologies and Quality of Reporting
Source: Front Cell Neurosci. 2020 Jun 16;14:164. doi: 10.3389/fncel.2020.00164 (PMC7309364; doi:10.3389/fncel.2020.00164)

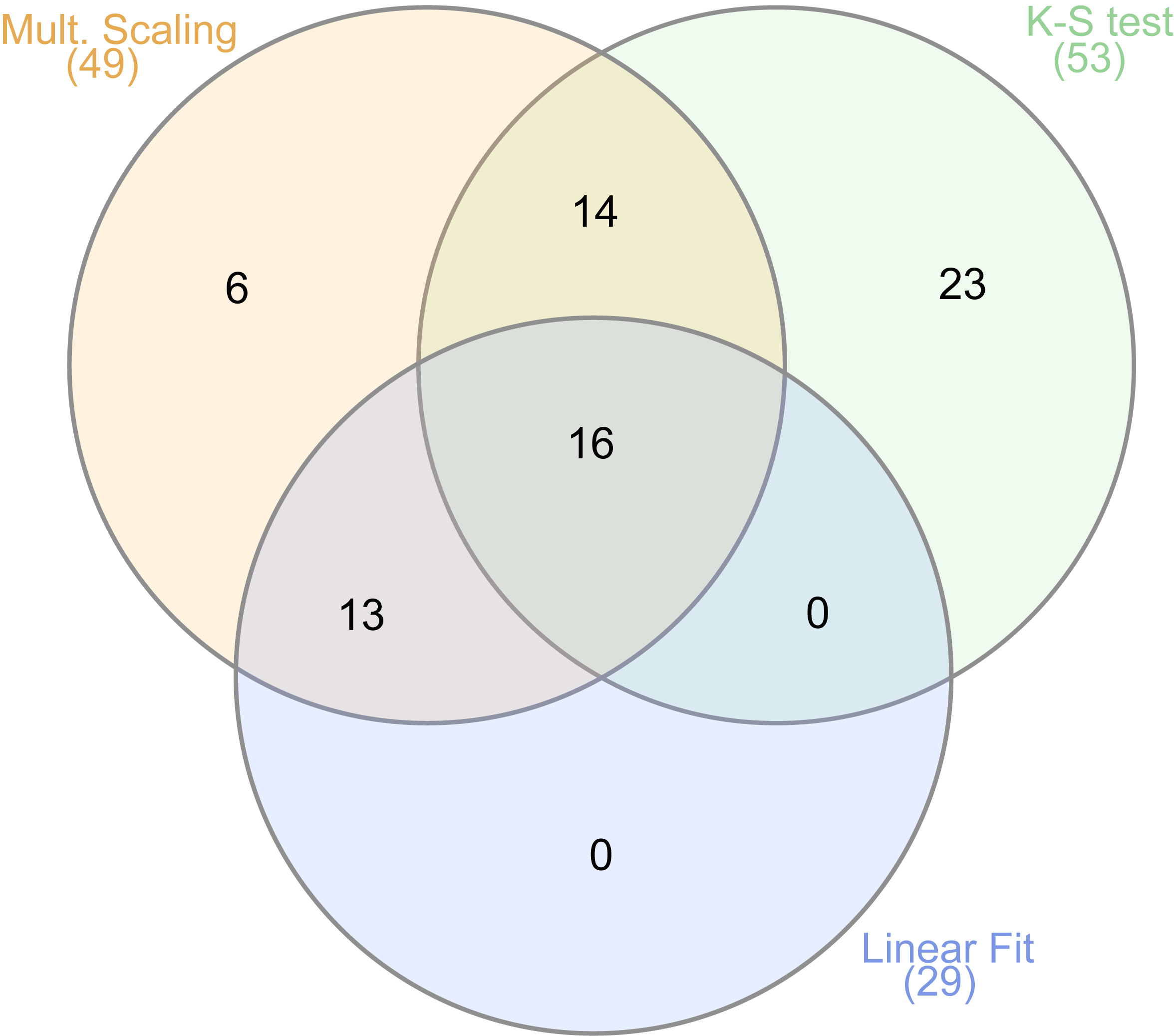

Supplement: Supplementary Figure 1 — Types of analysis for multiplicative scaling assessment. From the 168-studies sample, 49 (29%) mention observing multiplicative scaling of mPSC amplitudes. Within articles investigating multiplicative scaling, 14 (28.6%) describe using the Kolmogorov-Smirnov test for comparison of cumulative amplitude distributions, 13 (26.5%) employ linear regression of the ranked amplitudes, and 16 (32.6%) report using both approaches. A number of studies (23) mentioned the use of the Kolmogorov-Smirnov test without assessment of multiplicative scaling, but for simple group-comparison analysis. [file Image_1.tif]
