# Supplementary material for: The Synaptic Scaling Literature: A Systematic Review of Methodologies and Quality of Reporting
Source: Front Cell Neurosci. 2020 Jun 16;14:164. doi: 10.3389/fncel.2020.00164 (PMC7309364; doi:10.3389/fncel.2020.00164)
Supplement: Supplementary Table 1 — Country affiliation of the corresponding author. Percentages were calculated based on the total number of articles (n = 168). If an author had more than one country affiliation, the article counted for both of them; thus, the sum of percentages exceeds 100%. [file Table_1.DOCX]

Supplementary Material

| **Correspondence country** | **# Articles (%)** |
| --- | --- |
| USA | 113 (67.3) |
| Germany | 14 (8.3) |
| UK | 13 (7.7) |
| Canada | 6 (3.6) |
| China | 6 (3.6) |
| Italy | 4 (2.4) |
| Japan | 3 (1.8) |
| Finland | 2 (1.2) |
| France | 2 (1.2) |
| Israel | 2 (1.2) |
| Chile | 1 (0.6) |
| Korea | 1 (0.6) |
| Netherlands | 1 (0.6) |
| New Zealand | 1 (0.6) |
| Portugal | 1 (0.6) |
| Spain | 1 (0.6) |
| Switzerland/Spain | 1 (0.6) |
| Ukraine | 1 (0.6) |

**Supp Table 1. Country affiliation of the corresponding author.** Percentages were calculated based on the total number of articles (n=168). If an author had more than one country affiliation, the article counted for both of them; thus, the sum of percentages exceeds 100%.
