# Supplementary material for: The Synaptic Scaling Literature: A Systematic Review of Methodologies and Quality of Reporting
Source: Front Cell Neurosci. 2020 Jun 16;14:164. doi: 10.3389/fncel.2020.00164 (PMC7309364; doi:10.3389/fncel.2020.00164)
Supplement: Supplementary Table 2 — Main manipulations to induce synaptic scaling. The table shows the number of articles reporting the use of a given intervention, the percentages relative to the total number of articles included (n = 168), and their 95% confidence intervals. Spearman's correlation was used to estimate the ρ coefficient and p values for reporting trends over time. #Significantly correlated with time (α = 0.005 after Bonferroni correction for multiple comparisons). [file Table_2.DOCX]

| **Scaling-inducing intervention** | **# Articles (%) [95% C.I]** | **Trend over time (ρ)** | **p value** |
| --- | --- | --- | --- |
| TTX | 92 (54.8) [47.2, 62.1] | -0.0507 | 0.5141 |
| CNQX | 12 (7.1) [4.1, 12.1] | -0.2310 | 0.0026^#^ |
| APV | 20 (11.9) [7.8, 17.7] | -0.1826 | 0.0178 |
| Mix of TTX, CNQX or APV | 15 (8.9) [5.5, 14.2] | -0.1034 | 0.1824 |
| Bicuculline | 43 (25.6) [19.6, 32.7] | 0.1371 | 0.0765 |
| Picrotoxin | 13 (7.7) [4.6, 12.8] | 0.0333 | 0.6674 |
| Philanthotoxin-343 | 7 (4.2) [2.0, 8.3] | 0.1721 | 0.0257 |
| Nifedipine | 7 (4.2) [2.0, 8.3] | -0.1041 | 0.1794 |
| Visual deprivation | 18 (10.7) [6.8, 16.3] | -0.0334 | 0.6671 |
| Others | 41 (24.4) [18.5, 31.4] | -0.0495 | 0.5234 |

**Supp. Table 2. Main manipulations to induce synaptic scaling.** The table shows the number of articles reporting the use of a given intervention, the percentages relative to the total number of articles included (n=168), and their 95% confidence intervals. Spearman’s correlation was used to estimate the ρ coefficient and p values for reporting trends over time. ^#^ Significantly correlated with time (α = 0.005 after Bonferroni correction for multiple comparisons).
